# Sleep quality and mental health among Chinese nurses after the COVID-19 pandemic: A moderated model

**DOI:** 10.1371/journal.pone.0295105

**Published:** 2024-05-31

**Authors:** Yanyan Zhao, Fuzhi Liu, Pingzhen Lin, Zhuote Tu, Biyu Wu

**Affiliations:** 1 Department of Nursing, Quanzhou First Hospital, Quanzhou, China; 2 Department of Preventive Medicine, School of Health, Quanzhou Medical College, Quanzhou, China; Fondazione Policlinico Universitario Agostino Gemelli IRCCS, Universita’ Cattolica del Sacro Cuore, ITALY

## Abstract

**Introduction and aims:**

In the specialized nursing setting, nurses are susceptible to developing negative mental health issues. Such conditions among nurses can potentially result in unfavorable medical outcomes. Consequently, this study aims to explore the role of social support in regulating between sleep and mental health in nurses.

**Methods:**

A cross-sectional study was carried out in September 2022 on 1219 nurses in Quanzhou. The study comprised general demographic information and utilized various questionnaires, namely the Social Support Rate Scale (SSRS), Pittsburgh Sleep Quality Index Questionnaire (PSQI), Generalized Anxiety Disorder Questionnaire (GAD-7), and Patient Health Questionnaire-9 (PHQ-9). The data analysis was performed using t-tests, ANOVAs, Pearsons correlations and hierarchical regression analyses in SPSS software.

**Results:**

Results show that significant associations of sleep quality and social support with anxiety and depression. Simple slope analysis shows that under low levels of social support, sleep quality has a positive impact on anxiety(β = 0.598) and depression(β = 0.851), and the impact is significant. Under high levels of social support, sleep quality also has a positive impact on anxiety(β = 0.462) and depression(β = 0.578), but the impact is smaller. This indicates that as the level of social support increases, the positive predictive effect of sleep quality on anxiety and depression gradually diminishes.

**Conclusions:**

Social support has the potential to alter the impact of sleep quality on anxiety and depression. Therefore, healthcare policymakers need to focus on enhancing the level of social support and mitigating the impact of poor sleep on anxiety and depression.

## Introduction

Nursing is a profession that entails significant risks and a great deal of responsibility. Nurses are the primary caregivers, and their physical and mental well-being plays a crucial role in the quality of their work. Studies indicate that contemporary nurses are more susceptible to mental health issues, such as anxiety, depression, job burnout, sleep disturbances, and poor psychological resilience [1–3]. Among them, anxiety and depression are the most common psychological problems [4,5]. Moreover, The COVID-19 pandemic has resulted in a rapid increase in nursing workload that further exacerbates these psychological challenges [6]. In a systematic review and meta-analysis published in 2020, the prevalence of anxiety a nd depression among healthcare workers during the COVID-19 pandemic was found to be 23.2% and 22.8% [7]. Interestingly, in a subsequent study, the prevalence of anxiety and depression among healthcare workers was found to be 30.0% and 31.1% [8]. In a quantitative comparative cross-sectional study, the prevalence of anxiety between COVID and non-COVID intensive care units nurses was 36.5% and 27.3%, and the prevalence of depression was 21.2% and 9.1%, respectively [9]. It is imperative to address these issues effectively to ensure the well-being of nurses and promote quality care delivery. Without timely intervention, not only do the physical and mental health of nursing staff get affected, but it also increases the likelihood of adverse events occurring during clinical work, resulting in potential hazards to patient safety [10,11].

Sleep is a fundamental physiological requirement for individuals, including nurses [12]. However, due to various factors, nurses have become a vulnerable population with compromised sleep quality, characterized by insufficient sleep duration and poor sleep quality [13]. Sleep quality is defined as an individual’s self-satisfaction with all aspects of the sleep experience [14]. During the beginning of the COVID-19 pandemic in 2020, the prevalence of poor sleep quality among frontline nurses in Wuhan was 60% higher than that during regular prevention and control of the COVID-19 in China(43.7%) [15,16]. Sleep quality is affected by many factors, including individual factors(e.g., age, dietary nutrition, exercise, drug use), psychological and spiritual factors(e.g., anxiety, depression), and environmental factors(e.g., temperature, smell, blaze) [17–21]. The relationship between sleep quality and poor mental health outcomes, such as anxiety and depression, remains uncertain [22]. This implies that poor sleep quality may contribute to the development of anxiety and depression, which is supported by a significant body of literature [23–26]. Moreover, A recent study utilizing Mendelian randomization methodology has identified insomnia as a causal factor contributing to the development of anxiety [27].

Social support refers to the provision of assistance and protection to individuals, encompassing colleagues, managers, friends, and family members, which includes four attributes: emotional, instrumental, informational, and appraisal [28]. Social support has been found to have beneficial impacts on both physical and mental well-being and is widely recognized as crucial for nurses in effectively managing and addressing various stressors encountered within the workplace [29]. Numerous epidemiological studies have demonstrated a strong correlation between diminished levels of social support and the presence of anxiety and depression. Conversely, sufficient social support has been found to effectively mitigate the occurrence of these psychological phenomena [30,31]. Additionally, there have been reports indicating a negative correlation between social support and sleep quality, suggesting that individuals with higher levels of social support tend to experience better sleep quality [32]. The provision of effective social support has been proposed as a way to potentially improve sleep quality [33]. The presence of social support is believed to be a significant factor in understanding the potential association between sleep quality and anxiety and depression among nurses, as it is crucial for emotional regulation [34].

Despite numerous ongoing studies demonstrating the impact of sleep quality or social support on nurses’ mental health, there is a lack of research investigating whether social support can improve the role of sleep quality in anxiety and depression related to COVID-19. In order to address this inadequacy, the present study examined the psychological well-being of nurses and the factors that influence it, and explored whether social support affects the association between sleep quality and anxiety and depression. Furthermore, it aimed to clarify the varying levels of social support and their impact on anxiety and depression.

## Materials and methods

### Study design and setting

We conducted a cross-sectional study utilizing online self-reporting, and the survey employed a structured scale to gather quantitative data. The study took place at Quanzhou First Hospital of Fujian Province, which has a total of 1601 clinical nurses. The survey instrument was distributed to all nurses at the hospital via an online platform in September 2022.

### Participants

All clinical nurses registered in the Quanzhou First Hospital will be included as the study object. The inclusion criteria were: (1) All participants were over 18 years old. (2) Subjects in the study gave their informed consent to participate in this study. (3) Participants had more than one year of clinical work experience. Exclusion criteria were: (1) Nurses were under the age of 18. (2) There was personal or family history of mental health problems including anxiety and depression. (3) Participants were taking sleep medication.

### Survey instrument

The complete online survey, comprising socio-demographic data, measures of social support, sleep quality, and anxiety and depression scales, was employed to gather data from the research subjects.

### Demographics

The questionnaire includes basic demographic information such as age, gender, and marital status, as well as education.

### Social support

Social support was assessed using social support rate scale(SSRS) which comprised 10 self-reported items grouped into three domains: subjective support(four items), objective support(three items) and support availability(three items) [35]. The scale developed by Xiao has been extensively utilized in China, demonstrating strong validity and reliability(test-retest reliability R = 0.92). The total score is calculated by adding the scores of 10 items. A high level of social support is indicated by a score of more than 44 points, while a score between 23 and 44 points indicates a medium level of support, and a score of less than 23 points indicates a low level of support [36].

### Sleep quality

Pittsburgh Sleep Quality Index questionnaire(PSQI) is a self-report scale to assess sleep quality over the previous month [37]. The Chinese adaptation of the PSQI has been shown to possess reliability and validity when used within the Chinese population(the Cronbach α value was 0.83) [38]. This survey comprises of 19 items which can be scored from 0 to 3. The overall PSQI score ranges from 0 to 21. A score greater than 5 indicates poor sleep quality, while a score of 5 or less indicates good sleep quality [39].

### Anxiety

The 7-item Generalized Anxiety Disorder Questionnaire(GAD-7) is a 9-item self-report to assess anxiety which rated on 4 grades. The total score went from 0 to 21 [40]. The scale has been used previously in Chinese populations and has been found to have good reliability and validity(Cronbach α = 0.92, intraclass correlation = 0.83) [41,42]. The scoring criteria are as follows: A score of 0–4 is considered normal, while 5–9 indicates mild anxiety. Moderate anxiety falls within the range of 10–14, while a score of 15–21 suggests severe anxiety [43].

### Depression

Depression was measured with the Patient Health Questionnaire-9(PHQ-9) which contained 9 items [44]. Each item is rated on a 4-point scale. The total score ranged from 0 to 27. The evaluation criteria are the following: 0–4 indicates no depression, 5–9 mild depression, 10–14 moderate depression and 15–19 moderate to severe depression(RMSEA = 0.034, TLI = 0.985, CFI = 0.989) [45]. A score of 20–27 suggests the possibility of major depressive disorder.

### Data collection procedures

The Wenjuanxing platform is a specialized online survey tool that is extensively utilized in China. Users can import pre-designed questionnaires into the platform, which generates a unique link for distribution through various social media channels such as WeChat. We distributed the survey link to chief nurses and nurses in our database, requesting the former to disseminate the link through newsletters, emails, and other relevant communication channels within their networks. A covering letter accompanied the link, informing potential respondents about the survey’s purpose and timeframe. The survey’s landing page included links to the informed consent, frequently asked questions, and the survey itself. Before commencing the questionnaire, participants were obligated to review and agree to the informed consent in order to participate in the study. We used a limited survey approach, ensuring that each IP address could only submit one response. No personal data or IP addresses were gathered or retained, with data collection being anonymized.

### Ethics considerations

All research activities were conducted according to the Declaration of Helsinki procedures and received approval from the Institutional Review Board. The study was approved by the institution review board of Quanzhou First Hospital (NO. Quan Yi lun 2022217). All subjects provided their written informed consent to participate in this study.

### Data analysis

All data analysis was conducted using SPSS version 26.0. For the primary variables, a descriptive analysis and data centralization were carried out. This study examined associations between social support, sleep quality, anxiousness and depression using t-tests or ANOVAs and Pearsons correlations. Hierarchical regression analyses were conducted identifying the primary and interactive effects of social support and sleep quality on anxiety and depression. Covariates were at level 1 and social support were entered at level 2, while sleep quality was entered at level 3. The interaction term (SQ*SS) was then included at level 4. To assess the moderating effect, the researchers conducted simple slope tests to analyze the interaction pattern. We generated interaction effects plots using Excel templates obtained from online resources accessible at “Interpreting interaction effects” [46,47]. All tests are based on a two-sided test level of significance of 0.05.

## Results

### Demographics and descriptive analysis of the four key variables

A total of 1219 samples were collected in this study, with a recovery rate of 76.1%. The majority of participants were female (96.06%) with a mean age of 33.53±7.06 years old and the mean work experience of 11.55±8.08 years. 876(71.86%) of nurses were married and 51.52% have a college degree. 809(66.37%) nurses participated in the management of COVID-19 patients at the front line (Table 1). Social support, sleep quality, anxiety and depression, as shown in the Table 2, are influenced by demographic characteristics.

**Table 1:** Demographic information for sample.

Variables	Level	N	%
Age		M = 33.53, SD = 7.06	
Work experience		M = 11.55, SD = 8.08	
Gender	Male	48	3.94
	Female	1171	96.06
Education	College	628	51.52
	Undergraduate	591	48.48
Marital status	Married	876	71.86
	Others	343	28.14
Children	Yes	811	66.53
	No	408	33.47
Living with family members	Yes	737	60.46
	No	482	39.54
Frontline work	Yes	809	66.37
	No	410	33.63

**Table 2:** Social support, sleep quality, anxiety and depression based on demographic characteristics.

Variables	Level	N	Social Support	Sleep Quality	Anxiety	Depression
Age (years old)	<30	426	24.65±6.72	6.75±3.34	3.83±4.27	5.27±5.32
	30–40	598	27.52±6.27	6.91±3.58	4.73±4.74	5.93±5.83
	40–50	157	30.03±6.53	6.64±3.42	3.66±3.64	4.56±4.03
	>50	38	32.50±6.26	6.82±3.46	3.18±3.41	4.11±4.08
	*F*		40.65***	0.325	5.262***	3.938**
Gender	Male	48	24.77±7.79	5.52±3.70	3.33±4.24	4.33±4.92
	Female	1171	27.09±6.71	6.87±3.45	4.27±4.44	5.52±5.44
	*t*		-2.328*	-2.644**	-1.430	-1.481
Education	College	628	26.45±6.63	7.03±3.44	4.17±4.52	5.46±5.53
	Undergraduate	591	27.57±6.87	6.59±3.49	4.29±4.35	5.48±5.31
	*t*		-2.899**	2.206*	-0.481	-0.039
Marital status	Married	876	28.73±6.22	6.74±3.45	4.33±4.48	5.49±5.41
	Others	343	22.57±6.08	7.01±3.52	3.98±4.31	5.43±5.47
	*t*		15.656***	-1.212	1.247	0.175
Children	Yes	811	28.72±6.26	6.73±3.42	4.35±4.49	5.44±5.39
	No	408	23.57±6.44	6.99±3.56	4.00±4.37	5.53±5.50
	*t*		-13.430***	1.259	-1.311	0.297
Living with family members	Yes	737	25.94±6.77	6.89±3.48	4.36±4.56	5.64±5.58
	No	482	28.61±6.46	6.70±3.46	4.03±4.23	5.20±5.18
	*t*		-6.859***	0.918	1.252	1.395
Frontline work	Yes	809	25.91±6.79	6.90±3.53	5.40±5.33	4.14±4.35
	No	410	29.14±6.19	6.65±3.35	5.61±5.60	4.41±4.60
	*t*		-8.064***	1.199	-0.996	-0.666

* p < 0.05,

**p < 0.01,

***p < 0.001.

### Correlations

The correlations between social support, sleep quality, anxiety, and depression are presented in Table 3. Social quality was positively associated with anxiety(r = 0.436, P < 0.001) and depression(r = 0.485, P < 0.001). Social support was found to have a negative association with anxiety(r = -0.174, P < 0.001) and depression(r = -0.215, P < 0.001).

**Table 3:** Pearson correlations for social support, sleep quality, anxiety and depression.

Variables	Mean	SD	α	1	2	3	4
**1.social support**	27.00	6.77	0.958	1.000			
**2.sleep quality**	6.82	3.47	0.861	-0.176***	1.000		
**3.anxiety**	4.23	4.43	0.963	-0.174***	0.436***	1.000	
**4.depression**	5.47	5.43	0.945	-0.215***	0.485***	0.856***	1.000

***p < 0.001.

### The relationship between sleep quality, social support and anxiety

Social support regulates the relationship between sleep quality and anxiety, as evidenced in Table 4. The overall regression was statistically significant (Model 4:R^2^ = 0.208, F(df) = 52.92(6), p < 0.001). Sleep quality(β = 0.559) and frontline work(β = 0.514) are significantly correlated with anxiety, as indicated by a variance interpretation of 19.1%, was seen in level 2. In level 3, there was a significant positive correlation between sleep quality and anxiety. Conversely, social support was found to have a significant negative correlation with anxiety. The interpretation of the variance in these associations reached a level of 20.2%. The variance interpretation of the interaction term at level 4 experienced a 0.2% enhancement. The extent of social support exhibits considerable variation across different levels, and it functions as a mitigating factor in the relationship between sleep quality and anxiety. When social support is lower, the impact of sleep quality on anxiety is more pronounced. Conversely, when social support is higher, the influence of sleep quality on anxiety is reduced (Table 5 and S1 Fig).

**Table 4:** Linear hierarchical regression to examine the interactive effect of sleep quality and social support on anxiety.

Predictor		β	Std.β	t	95%CI for β		VIF	Adjust R square	ΔF	P
					Lower	Upper				
Level 1	Gender	0.962	0.042	1.458	-0.332	2.256	1.022	0.003	1.41	0.239
	Age	-0.023	-0.037	-1.200	-0.061	0.015	1.159			
	Frontline work	0.352	0.038	1.222	-0.213	0.917	1.149			
Level 2	Gender	0.159	0.007	0.266	-1.010	1.327	1.028	0.191	285.74	0.000***
	Age	-0.021	-0.034	-1.210	-0.055	0.013	1.159			
	Frontline work	0.514	0.055	1.983*	0.006	1.023	1.151			
	SQ	0.559	0.437	16.904***	0.494	0.623	1.008			
Level 3	Gender	0.242	0.011	0.409	-0.919	1.404	1.029	0.202	17.53	0.000***
	Age	-0.002	-0.003	-0.099	-0.037	0.033	1.241			
	Frontline work	0.646	0.069	2.490*	0.137	1.156	1.168			
	SQ	0.533	0.418	15.978***	0.468	0.599	1.042			
	SS	-0.076	-0.116	-4.187***	-0.111	-0.040	1.167			
Level 4	Gender	0.220	0.010	0.372	-0.940	1.380	1.030	0.204	4.22	0.040*
	Age	-0.001	-0.002	-0.064	-0.036	0.034	1.242			
	Frontline work	0.681	0.073	2.622**	0.172	1.191	1.173			
	SQ	0.530	0.415	15.898***	0.465	0.596	1.044			
	SS	-0.080	-0.122	-4.391***	-0.116	-0.044	1.181			
	SQ*SS	-0.010	-0.053	-2.053*	-0.019	0.000	1.016			

*p < 0.05,

**p < 0.01,

***p < 0.001,

SQ: Sleep quality, SS: Social support.

**Table 5:** Results of simple slope test of the effect of sleep quality on anxiety at different groups of social support.

Group	Simple slope	*t*	*P*
Low SS	0.598	13.664	0.000***
High SS	0.462	10.082	0.000***

***p < 0.001,

SS: Social support.

### The relationship between sleep quality, social support and depression

The relationship between sleep quality and depression is regulated by social support, as demonstrated in Table 6. The overall regression was statistically significant (Model 4: R^2^ = 0.266, F(df) = 73.22(6), p < 0.001). The results indicate that sleep quality (β = 0.760) and frontline work (β = 0.634) are significantly associated with anxiety, accounting for 23.7% of the variance at level 2. At level 3, there is a significant positive correlation between sleep quality and depression, while social support is found to have a significant negative correlation with depression. The interpretation of the variance in these associations reaches 25.5%. Additionally, the interaction term at level 4 shows a 0.7% enhancement in variance interpretation. When social support is at a low level, the impact of sleep quality on depression is significantly greater. When social support is at a high level, the impact of sleep quality on depression is relatively smaller (Table 7 and S2 Fig).

**Table 6:** Linear hierarchical regression to examine the interactive effect of sleep quality and social support on depression.

Predictor		β	Std.β	t	95%CI for β		VIF	Adjust R square	ΔF	P
					Lower	Upper				
Level 1	Gender	1.310	0.047	1.625	-0.272	2.892	1.022	0.003	2.11	0.097
	Age	-0.047	-0.061	-1.966*	-0.093	0.000	1.159			
	Frontline work	0.413	0.036	1.174	-0.277	1.104	1.149			
Level 2	Gender	0.218	0.008	0.309	-1.17	1.607	1.028	0.237	95.66	0.000***
	Age	-0.044	-0.057	-2.106*	-0.084	-0.003	1.159			
	Frontline work	0.634	0.055	2.058*	0.030	1.239	1.151			
	SQ	0.76	0.486	19.348***	0.682	0.837	1.008			
Level 3	Gender	0.347	0.012	0.495	-1.026	1.719	1.029	0.255	29.57	0.000***
	Age	-0.014	-0.018	-0.660	-0.056	0.028	1.241			
	Frontline work	0.837	0.073	2.727***	0.235	1.439	1.168			
	SQ	0.721	0.461	18.265***	0.643	0.798	1.042			
	SS	-0.116	-0.145	-5.438***	-0.158	-0.074	1.167			
Level 4	Gender	0.299	0.011	0.429	-1.067	1.665	1.030	0.262	13.70	0.000***
	Age	-0.013	-0.016	-0.599	-0.054	0.029	1.242			
	Frontline work	0.911	0.079	2.977**	0.310	1.511	1.173			
	SQ	0.714	0.457	18.188***	0.637	0.792	1.044			
	SS	-0.125	-0.156	-5.835***	-0.167	-0.083	1.181			
	SQ*SS	-0.020	-0.092	-3.702***	-0.031	-0.010	1.016			

*p < 0.05,

**p < 0.01,

***p < 0.001,

SQ: Sleep quality, SS: Social support.

**Table 7:** Results of simple slope test of the effect of sleep quality on depression at different groups of social support.

Group	Simple slope	*t*	*P*
Low SS	0.851	29.745	0.000***
High SS	0.578	8.155	0.000***

***p < 0.001, SS: Social support.

## Discussion

In various systematic reviews and meta-analyses, healthcare professionals have reported significant mental health issues, including elevated levels of anxiety and depression. The study discovered higher levels of anxiety(4.23±4.43) and depression(5.47±5.42) compared to previous outcomes(anxiety:4.10±4.34; depression:5.09±5.62) of the analogous study [48]. One possible explanation for this phenomenon could be the notable surge in COVID-19 cases within the Quanzhou city population in March 2022. Consequently, clinical nurses in this context are required to deliver heightened care to these patients, resulting in an augmented workload and increased work-related stress. Hence, disregarding the indications of distress and depression exhibited by nursing professionals can potentially exacerbate personal physical and emotional strain, while also contributing to substandard patient care and heightened work demands within the organization [49].

This study examined the sleep patterns of nurses in the aftermath of the COVID-19 pandemic. The findings in the paper revealed a positive association between the sleep quality of nurses and their levels of anxiety(β = 0.436) and depression(β = 0.485). This suggests that nurses with poorer sleep quality tend to exhibit higher scores of anxiety and depression. Furthermore, the results of the linear hierarchical regression analysis indicated that even after accounting for factors such as age, gender, and frontline work variables, nurses’ sleep quality remained a significant predictor of anxiety and depression. Despite taking into account the impact of social support on nurses, the quality of sleep can still have a positive influence on levels of anxiety and depression. These findings align with previous research in this area [50–53]. Furthermore, the above epidemiological research has indicated a positive correlation between sleep problems and anxiety and depression. The experimental and mechanistic studies have clarified that issues with sleep quality may contribute to the development of anxiety and depression through the nervous or endocrine systems [54–56].

The results of this study demonstrate a negative correlation between social support and anxiety and depression. Specifically, individuals with higher levels of social support exhibit lower levels of anxiety and depression, which similar with previous research findings [57–60]. It is conceivable that the negative emotional challenges, such as anxiety and depression, faced by nurses amid the COVID-19 pandemic play a role in this correlation, as indicated by the results of our research. Nurses who have access to robust social support networks, including family, friends, or colleagues, are able to effectively manage negative emotions and alleviate the severity of anxiety and depression. Conversely, nurses with limited social support are more likely to face the challenges of the pandemic in isolation, without the opportunity to regulate their emotions through social interaction, resulting in heightened levels of anxiety and depression [61,62]. Furthermore, the study reveals that sleep quality has a positive predictive impact on anxiety and depression, as supported by the regulatory model. However, this positive predictive effect of sleep quality on anxiety and depression diminishes as social support increases. Additionally, it is observed that nurses with high levels of social support experience lower levels of anxiety and depression compared to those with low levels of social support, regardless of their sleep quality. This suggests that higher levels of social support may contribute to improved sleep quality, consequently reducing the occurrence of negative emotions such as anxiety and depression. This could be attributed to the increased support nurses receive from organizations and their social networks as their level of social support increases [63,64].

## Limitations

There are still several limitations to this study. Firstly, this study employed a cross-sectional methodology, which limits its ability to establish a causal link between sleep quality and anxiety and depression. Additionally, the sample was restricted to nurses from a single hospital, necessitating further investigation among diverse populations to validate the findings. In addition, the study’s sample consisted primarily of females, which could decrease the generalisability of our results. Thirdly, our data collection method utilises self-reported questionnaires which may produce information bias. This study employed screening scales to assess the sleep quality, as well as the presence and severity of anxiety and depression. These measures were not intended to provide a clinically definitive diagnosis. For example, The assessment of sleep quality is predominantly reliant on subjective measures, and its quantification lacks consistency with existing metrics. However, in the future, the utilization of objective evaluations such as wearable devices may enable the continuous measurement of sleep. Ultimately, physical activity plays a significant role in influencing both the quality of sleep and mental well-being. However, the current study did not examine the participants’ level of physical exercise, thus failing to account for this potentially confounding variable. This omission may result in inconsistencies in findings, emphasizing the importance of including exercise levels as an exclusion criterion in forthcoming research investigations.

## Conclusions

The quality of sleep has been found to have a positive impact on the levels of anxiety and depression experienced by nurses. Additionally, it has been observed that with a certain level of social support, sleep quality can also contribute to a reduction in mental health issues among nurses. Consequently, it is recommended that nursing managers adopt a scientific and humanized approach to management, which may include implementing flexible scheduling to enhance the working environment for nurses, alleviating work-related stress, and offering accessible social assistance, such as psychological counseling, when needed. These measures aim to mitigate the occurrence of psychological problems among nurses.

## Supporting information

S1 Raw data (XLSX)

S1 Fig Interaction effects of sleep quality and social support in predicting anxiety. (TIF)

S2 Fig Interaction effects of sleep quality and social support in predicting depression. (TIF)

S3 Fig Histograms of the standardized residuals of the anxiety model. (TIF)

S4 Fig Normal P-P plot of regression standardized residual of the anxiety model. (TIF)

S5 Fig Histograms of the standardized residuals of the depression model. (TIF)

S6 Fig Normal P-P plot of regression standardized residual of the depression model. (TIF)

## Abbreviations

ANOVA: Analysis of Variance
GAD-7: the 7-item Generalized Anxiety Disorder Questionnaire
PHQ-9: Patient Health Questionnaire-9
PSQI: Pittsburgh Sleep Quality Index
SSRS: Social Support Rate Scale
