# Supplementary figures and images for: Sleep quality and mental health among Chinese nurses after the COVID-19 pandemic: A moderated model
Source: PLoS One. 2024 May 31;19(5):e0295105. doi: 10.1371/journal.pone.0295105 (PMC11142611; doi:10.1371/journal.pone.0295105)

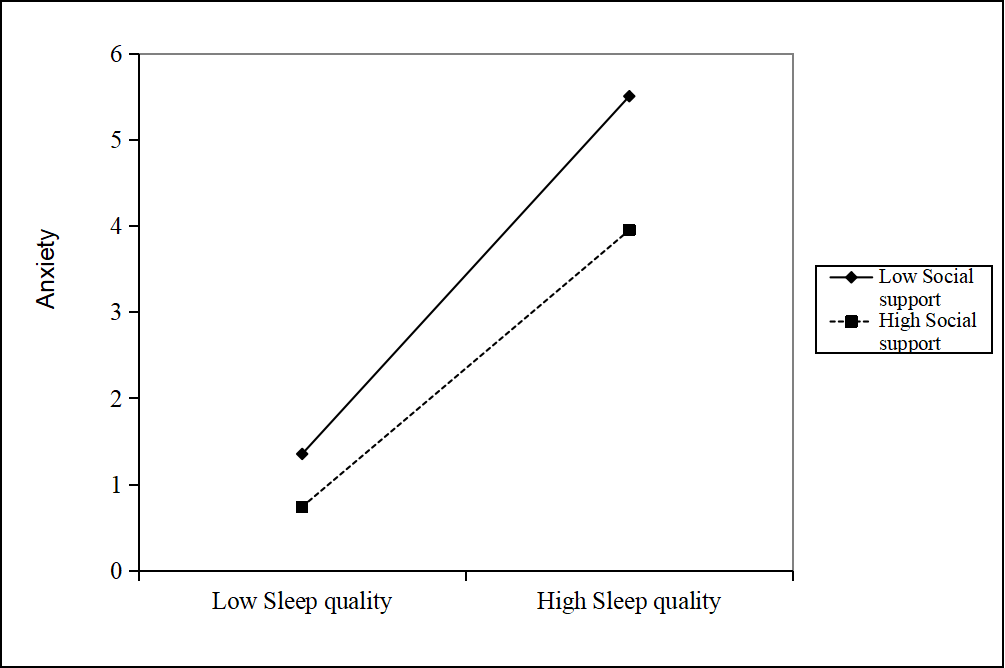

Supplement: S1 Fig — (TIF) [file pone.0295105.s002.tif]

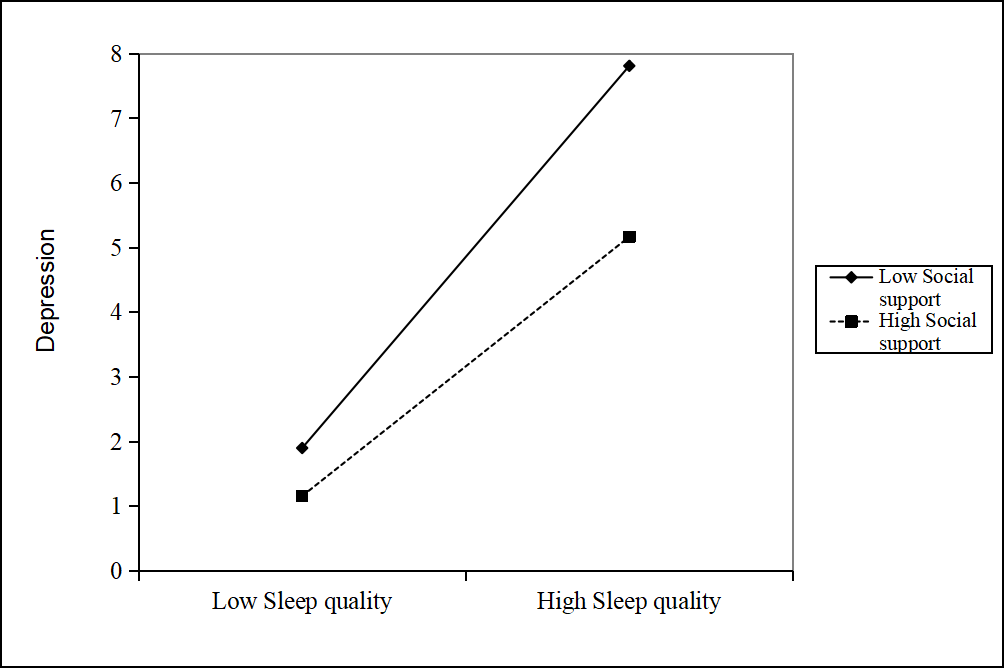

Supplement: S2 Fig — (TIF) [file pone.0295105.s003.tif]

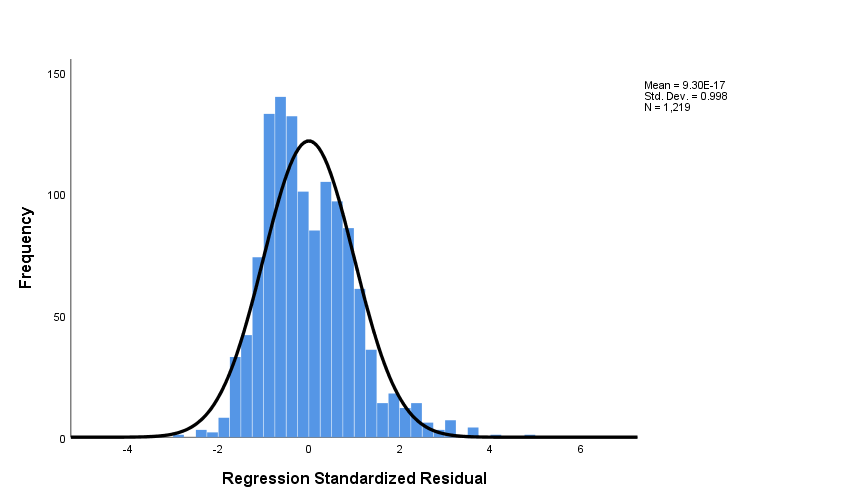

Supplement: S3 Fig — (TIF) [file pone.0295105.s004.tif]

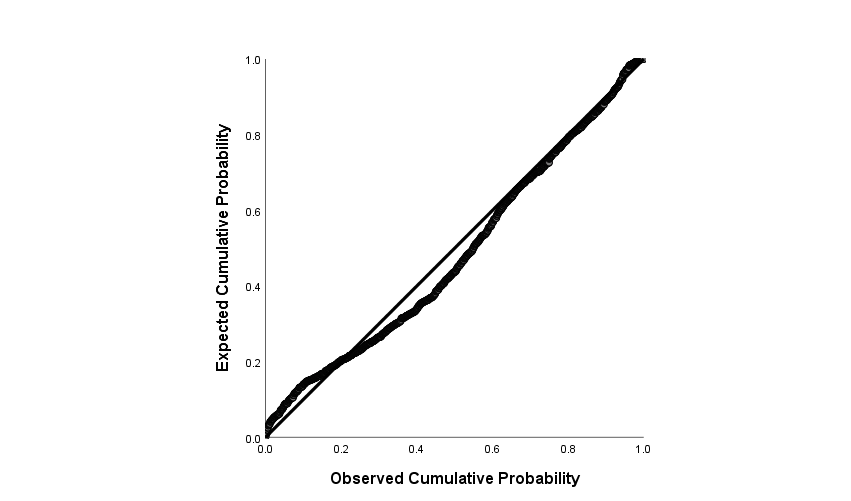

Supplement: S4 Fig — (TIF) [file pone.0295105.s005.tif]

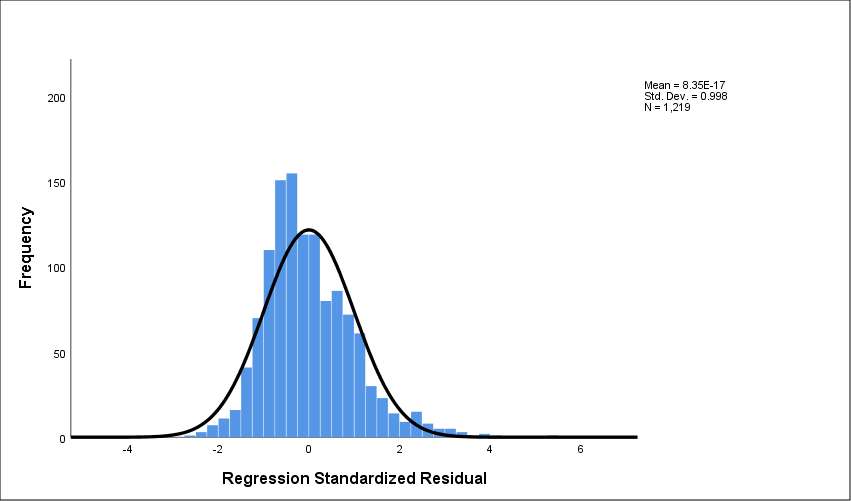

Supplement: S5 Fig — (TIF) [file pone.0295105.s006.tif]

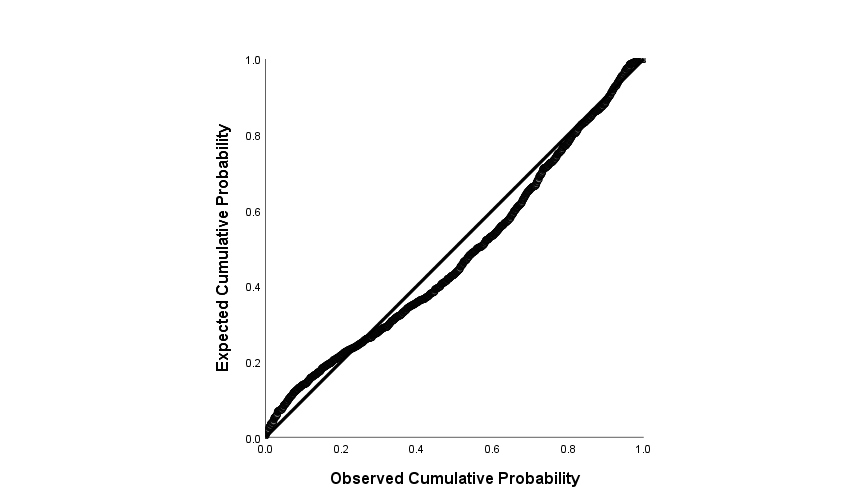

Supplement: S6 Fig — (TIF) [file pone.0295105.s007.tif]
